# Association of progression-free survival with patient-reported outcomes and survival: results from a randomised phase 3 trial of panitumumab

**DOI:** 10.1038/sj.bjc.6604053

**Published:** 2007-11-27

**Authors:** S Siena, M Peeters, E Van Cutsem, Y Humblet, P Conte, E Bajetta, D Comandini, G Bodoky, G Van Hazel, T Salek, M Wolf, G Devercelli, M Woolley, R G Amado

**Affiliations:** 1 Ospedale Niguarda Ca' Granda, Milan, Italy; 2 Ghent University Hospital, Ghent, Belgium; 3 University Hospital Gasthuisberg, Leuven, Belgium; 4 St-Luc University Hospital, Brussels, Belgium; 5 UO Oncologia Medica – Centro Oncologico Modenese COM, Modena, Italy; 6 Istituto per lo Studio a la Cura dei Tumori, Milan, Italy; 7 Ospedale San Martino, Genova, Italy; 8 Szent László Hospital, Budapest, Hungary; 9 Clinical Trials Centre, West Perth WA, Australia; 10 National Cancer Institute, Bratislava, Slovak Republic; 11 Amgen Inc., Thousand Oaks, CA, USA

**Keywords:** panitumumab, patient-reported outcomes, quality of life, disease progression, symptom, improvement

## Abstract

In a randomised phase 3 trial, panitumumab significantly improved progression-free survival (PFS) in patients with refractory metastatic colorectal cancer (mCRC). This analysis characterises the association of PFS with CRC symptoms, health-related quality of life (HRQoL), and overall survival (OS). CRC symptoms (NCCN/FACT CRC symptom index, FCSI) and HRQoL (EQ-5D) were assessed for 207 panitumumab patients and 184 best supportive care (BSC) patients who had at least one post-baseline patient-reported outcome (PRO) assessment. Patients alive at week 8 were included in the PRO and OS analyses and categorised by their week 8 progression status as follows: no progressive disease (no PD; best response of at least stable disease) *vs* progressive disease (PD). Standard imputation methods were used to assign missing values. Significantly more patients were progression free at weeks 8–24 with panitumumab *vs* BSC. After excluding responders, a significant difference in PFS remained favouring panitumumab (HR=0.63, 95% CI=0.52–0.77; *P*<0.0001). At week 8, lack of disease progression was associated with significantly and clinically meaningful lower CRC symptomatology for both treatment groups and higher HRQoL for panitumumab patients only. Overall survival favoured no PD patients *vs* PD patients alive at week 8. Lack of disease progression was associated with better symptom control, HRQoL, and OS.

Colorectal cancer (CRC) is the third most common cancer worldwide, with more than one million new diagnoses each year, and more than 500 000 deaths annually (Parkin *et al*, 2005). Despite advancements in treatment for this disease which include cytotoxic agents and targeted therapies, most patients eventually progress, become symptomatic, and succumb to their disease (de Gramont *et al*, 2000; Douillard *et al*, 2000; Saltz *et al*, 2000; Rothenberg *et al*, 2003; Cunningham *et al*, 2004; Hurwitz *et al*, 2004; Saltz *et al*, 2004; Tournigand *et al*, 2004).

Progression-free survival (PFS) has been shown to correlate with overall survival (OS) in the first-line setting for metastatic colorectal cancer (mCRC)(Tournigand *et al*, 2004). Since disease progression is a consequence of tumour growth, and it is often associated with symptomatic progression, using PFS as a surrogate end point in CRC trials is also likely to predict clinical benefit in the setting of refractory disease. However, the role of PFS as a predictor of OS in later lines of treatment for mCRC has not yet been demonstrated.

In mCRC, monoclonal antibodies that target the epidermal growth factor receptor (EGFR) have demonstrated efficacy (Cunningham *et al*, 2004; Van Cutsem *et al*, 2007a, 2007b). Currently, two anti-EGFR antibodies are approved for the treatment of mCRC, panitumumab and cetuximab (Yang *et al*, 1999; Yarden and Sliwkowski, 2001; Foon *et al*, 2004). Panitumumab is a fully human monoclonal antibody that inhibits ligand binding (e.g., EGF and TGF-*α*) and associated cellular processes that lead to tumour growth, cell survival, and metastases. A phase 3 randomised-controlled trial of panitumumab plus best supportive care (BSC) *vs* BSC alone in patients with mCRC who had progressed after standard therapy demonstrated that panitumumab plus BSC was well tolerated and significantly improved PFS (Van Cutsem *et al*, 2007a).

The clinical benefit of delaying tumour progression (i.e., PFS) was not fully explored in the primary analysis of this study. From the primary analysis of patient-reported outcomes (PROs), no significant differences in CRC symptoms and health-related quality of life (HRQoL) were seen between the two treatment groups (Van Cutsem *et al*, 2007a). Potential HRQoL and CRC symptom benefits associated with panitumumab therapy are important aspects of clinical benefit, particularly in a chemorefractory population with progressing disease. In addition, OS is a recognised standard end point in this setting. In this report, exploratory analyses were conducted that assessed the association between PFS and patient-reported CRC symptoms and HRQoL, as well as OS.

## PATIENTS AND METHODS

### Patients and study design

**Figure 1:**
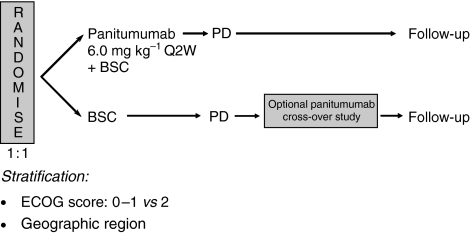
Phase 3 study schema.

This phase 3 multicentre, open-label randomised-controlled trial evaluated the efficacy and safety of panitumumab plus BSC (panitumumab) compared with BSC alone (BSC) given every 2 weeks in patients with mCRC that had progressed on prior fluoropyrimidine, irinotecan, and oxaliplatin (Figure 1) (Van Cutsem *et al*, 2007a). Relevant inclusion criteria were pathologic diagnosis of metastatic colorectal adenocarcinoma, radiologic documentation of disease progression during or within 6 months following the last administration of fluoropyrimidine, irinotecan, and oxaliplatin, prior exposure to prespecified doses of irinotecan and oxaliplatin, and two or three prior chemotherapy regimens. Both criteria of disease progression and dose intensity were retrospectively centrally confirmed. Additional details of the study design and key eligibility criteria are reported in the primary publication (Van Cutsem *et al*, 2007a).

Objective tumour response was assessed by central radiology review using modified RECIST criteria (Therasse *et al*, 2000) at specified time points from weeks 8 to 48, and every 3 months thereafter until disease progression. Responses were confirmed not less than 4 weeks after response criteria were first met. Tumour response, including stable disease, was evaluated at the first scheduled assessment (week 8). At the discretion of the investigator, patients could be evaluated for radiographic tumour assessment after developing symptoms consistent with disease progression. Best supportive care patients determined by investigator to have disease progression were allowed to receive panitumumab under a separate cross-over study (Van Cutsem *et al*, 2007a, 2007b).

### HRQoL and CRC symptomatology assessments

Patient-reported outcome assessments were taken at baseline, every 2 weeks or monthly during the treatment phase of the study, and at the 30-day safety follow-up visit (Table 1). CRC symptomatology was measured using the NCCN FACT Colorectal Symptom Index (FCSI) and HRQoL was measured using the EQ-5D Health Index Scale, the EQ Visual Analog Scale (VAS), and two global health items from the Global Quality of Life Scale of the European Organizations for the Research and Treatment of Cancer Quality of Life Questionnaire – Core (EORTC QLQ-C30).

The FCSI was derived as a set of brief, clinically relevant CRC symptoms for assessing symptomatic response. It comprises of the most important symptoms associated with CRC including pain, energy, diarrhoea, nausea, stomach swelling or cramps, appetite, and weight loss, and more general aspects of HRQoL such as ability to enjoy life and contentedness with QoL (Cella *et al*, 2003). The nine-item FCSI is scored to produce a total score. Linear transformation was used to standardise the raw score, so that the total score ranges from 0 (severely symptomatic on all symptoms assessed) to 100 (symptom-free on all symptoms assessed). For the FCSI, the minimal clinical important difference (MCID) was defined as a change in score of 4 points or more (in a scale of 0–100) (Mathias *et al*, 2006). The FCSI was specifically developed to be used in CRC research and designed to be sensitive to symptomatic progression as experienced by patients with metastatic disease (Cella *et al*, 2003). The relevance of these specific symptoms also correlates well with routine clinical observations of those engaged in the care of these patients.

The EQ-5D Health Index Scale provides a preference-weighted assessment of overall QoL across five dimensions that include mobility, self-care, usual activity, pain or discomfort, and anxiety or depression. Each dimension has three possible outcomes (no problems, moderate problems, and extreme problems), with a total score ranging from 0 corresponding with death to 1 corresponding with perfect health (Walters and Brazier, 2005). Estimates for the MCID for the EQ-5D Index include a change in score of 0.074 points (Osoba *et al*, 1998) and 0.08 points or more (Osoba *et al*, 1998; Mathias *et al*, 2006).

The EQ-5D Visual Analog Scale (VAS) provides an assessment of current health status on a vertical scale and ranges from 0 to 100 with 0 representing worst imaginable health state and 100 representing best imaginable health state (Brooks *et al*, 2003). For the EQ-5D VAS, the MCID was defined as a change in score of 5.48 points or more (Mathias *et al*, 2006).

The EORTC QLQ-C30 is a validated HRQoL 30-item instrument (Aaronson *et al*, 1993). In this study, the Global Health Status/QoL Scale of the EORTC QLQ-C30 was used. It consists of two items measuring overall health status and overall QoL. Linear transformation is used to standardise the raw score into a scale ranging from 0 to 100 with higher scores indicating better overall QoL (Fayers *et al*, 1995). For the Global Health Status/QoL Scale, the MCID was defined as a change in score of 7.07 points or more (Mathias *et al*, 2006).

### Statistical analysis

The primary study end point was PFS by blinded central radiology assessment. The primary analysis of PFS included tumour assessments after crossover to panitumumab for BSC patients if disease progression was not centrally confirmed while the patient received BSC alone. Secondary end points included best objective response by blinded central review (prespecified for testing), OS, and PROs as described above. To assess whether the treatment differences in PFS were due to patients with an objective response, a post-hoc sensitivity analysis of PFS that removed responding patients in the panitumumab group (i.e., patients with a best response of at least partial response) was conducted to evaluate the contribution of non-responding patients on the treatment effect with panitumumab. Statistical analyses for the primary and secondary end points have been described previously (Van Cutsem *et al*, 2007a, 2007b).

The objective of the analyses presented herein was to evaluate the association between delaying tumour progression (i.e., PFS) and CRC symptoms, HRQoL, and OS. For PRO analyses (i.e., CRC symptoms and HRQoL), patients were included if they had at least one post-baseline PRO assessment (the PRO all enrolled analysis set). Additionally, to help reduce bias, patients who died prior to week 8 were excluded from the PRO analysis. Patients were categorised into one of two groups based on progression status as of week 8 as follows: (1) patients who were progression free (i.e., had a best confirmed response of at least stable disease); or (2) patients with tumour progression (i.e., had less than stable disease).

*t*-Tests and least-squares estimates were calculated for differences in PRO measures at weeks 4, 8, 12, and 16 controlling for baseline score by progression status as of week 8 (no tumour progression *vs* tumour progression) within each treatment arm. For each treatment group, descriptive statistics of point estimates of scores over time for all instruments and of the difference in scores between the two tumour progression groups over time for all instruments were summarised.

Missing PRO data were imputed using the following two methods: last value carried forward (LVCF) method and a slope method (Fairclough, 2002; Fielding *et al*, 2006). For the LVCF method, missing observations were replaced with the last observed value carried forward for reasons other than death or zero value carried forward at death. Additional rules for the LVCF method were as follows: (1) patients missing values in between two observed values had the earlier observed value carried forward unless the subsequent observed value occurred after disease progression, in which case the later observed value was carried backward; and (2) if the baseline data were missing then the missing data were imputed using the first observation carried backwards.

The slope method applied forward linear interpolation of observed data to impute missing data. Time was measured relative to randomisation with observed data assigned to the day it was obtained, and the day of a missed assessment is assigned to the day the assessment was scheduled per protocol. The predicted value from a linear regression of observed data prior to a missed assessment was used to impute a value at the time of the missed assessment. Prior imputed values were not used to impute subsequent values. If there was only one observed value prior to a missed assessment, then the last value was carried forward to the missed assessment. If the first scheduled assessment was missing, then it was not imputed.

For OS, within each treatment group, survival was examined among patients surviving to at least week 8 according to their progression status as of week 8. A Cox regression model was used to examine the correlation between time to radiologic progression (TTP) and time to death (TTD) among all patients. Time to radiologic progression was defined as the time from randomisation to radiologic disease progression per modified-RECIST by blinded central review. Patients who died without radiologic progression were censored at their last radiologic assessment of TTP. The model included indicators for the randomisation factors (ECOG performance score and geographic region) and a time-dependent covariate for radiologic progressive disease (PD).

## RESULTS

### Demographics

In this phase 3 trial, 463 patients were randomised, 231 patients to panitumumab and 232 patients to BSC (Van Cutsem *et al*, 2007a). Median follow-up time for survival (enrollment to data cutoff for analysis) for all patients was 72 weeks (range=52–113). In the BSC group, 176 (76%) patients received panitumumab under the crossover study (Van Cutsem *et al*, 2007b).

In the all randomised analysis set, 22 (10%) of panitumumab patients had a partial response and 62 (27%) patients had stable disease. Of the BSC patients, no patients had an objective response and 23 (10%) patients had stable disease. Disease control (the sum of objective response and stable disease rates) was seen in 84 (36%) panitumumab patients and 23 (10%) BSC patients.

For the PRO analyses, 207 (90%) panitumumab patients and 184 (79%) BSC patients had at least one post-baseline PRO assessment and comprised the PRO All Enrolled analysis set (Table 2). As expected from the higher rate of discontinuation because of disease progression in the BSC group, a higher percentage of panitumumab patients had evaluable PRO data compared with the BSC group from week 4 onwards. At week 4, EQ-5D data were available for 91% of panitumumab patients and 70% of BSC patients; by week 16, data were available for 30% and 4% of patients, respectively. The proportion of patients with available data for the FCSI at weeks 4 and 16 for each treatment group were similar as that for the EQ-5D at those respective time points.

### Contribution of stable disease on PFS

**Figure 2:**
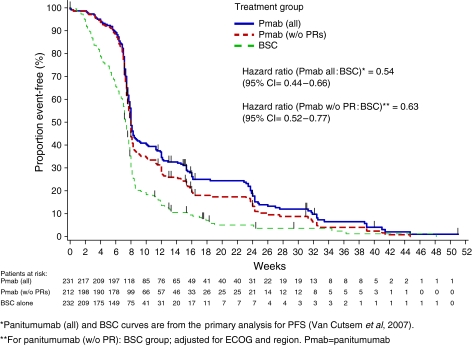
Sensitivity analysis of progression-free survival (PFS) with and without responders in the panitumumab group.

To evaluate the importance of achieving stable disease in this population, an analysis was conducted where panitumumab patients who had an objective response were excluded from the primary analysis of PFS (all randomised patients, panitumumab *vs* BSC). A significant difference in PFS remained for panitumumab over BSC (hazard ratio=0.63, 95% CI=0.52–0.77; *P*<0.0001) (Figure 2). These data indicated that improvement in PFS was not derived solely from the subset of patients with a best response of partial response. Indeed, 80% of the overall treatment effect could be accounted for by panitumumab patients without an objective response, specifically by patients with stable disease.

### Association between PFS and disease-related symptoms and HRQoL

Patient-reported outcomes were evaluated in each treatment group for patients alive at week 8 and stratified by tumour progression status as of week 8. For both the FCSI and EQ-5D Health Index, among panitumumab patients, those who were progression free at week 8 maintained mean scores throughout treatment while those with tumour progression at week 8 had a steady decline in the mean scores throughout treatment (data not shown).

**Figure 3:**
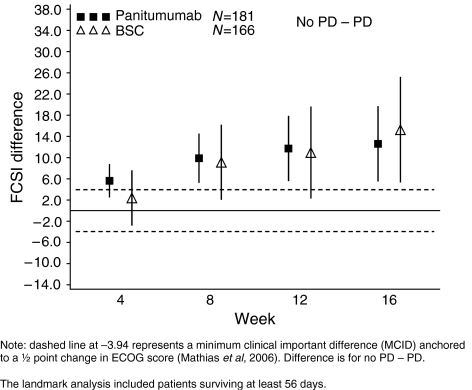
Mean FACT-FCSI difference (95% CI) in scores by progression status at week 8 (central review, patient-reported outcome (PRO) all enrolled, landmark analysis).

The difference in the FCSI scores between patients who were progression free (no PD) and those with progression (PD) at week 8 was evaluated in both treatment groups (Figure 3). For both panitumumab or BSC groups, achieving at least stable disease at week 8 (i.e., being progression free) was associated with significant and clinically meaningful improvement in control of disease-related symptoms at all time points (weeks 4, 8, 12, and 16) (Figure 3). The lower limits of the 95% confidence intervals excluded 0 (i.e., statistical significance) and the point estimates exceeded the MCID of −4 points (i.e., clinically meaningful) at all time points for the panitumumab group and at weeks 8 to16 for the BSC group.

**Figure 4:**
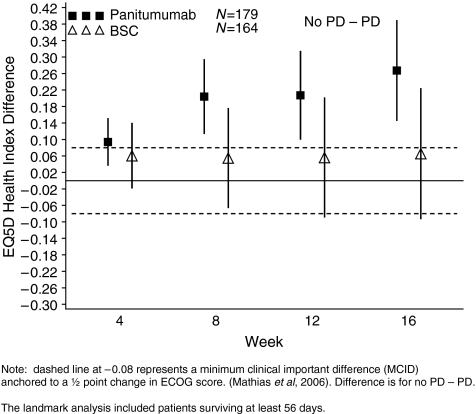
Mean EQ-5D Health Index Difference (95% CI) in scores by progression status at week 8 (central review, patient-reported outcome (PRO) all enrolled, landmark analysis).

For the EQ-5D Health Index, the difference in scores for panitumumab patients alive at week 8 who did not have tumour progression minus those with progression was significantly and clinically meaningful at all time points (Figure 4). For BSC patients, the difference in scores between the tumour progression groups was not significant or clinically meaningful (Figure 4). Lack of disease progression with panitumumab for patients alive at week 8 was associated with better HRQoL.

Results for the FCSI and EQ-5D for all treatment groups were similar regardless of imputation method. Additionally, similar results for panitumumab and BSC patients stratified by tumour progression status were observed for the EQ-5D VAS and EORTC Global Scale (data not shown).

### Association of tumour progression status on OS in the panitumumab patients

**Figure 5:**
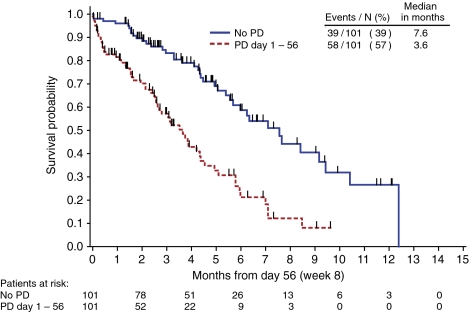
Overall survival (OS) in subset of panitumumab patients alive at week 8 and categorised by tumour progression status at week 8.

Panitumumab patients who were alive at week 8 without PD had a median TTD of 7.6 months (Figure 5). For panitumumab patients alive with a PD at week 8, the median TTD was 3.6 months. For BSC patients who were alive at week 8, those without PD had a median TTD of 8.6 months *vs* those with PD who had a median TTD of 4.3 months (data not shown).

## DISCUSSION

This exploratory analysis was conducted to evaluate the association of tumour progression status with HRQoL. Here, we first evaluated the contribution of stable disease on the overall effect of panitumumab on PFS. These analyses revealed that 80% of the treatment effect of panitumumab on PFS was retained after removing patients that responded to the drug. These results indicate that the treatment benefit with panitumumab is obtained in patients with disease stabilisation. These results are of interest given that patients in this trial had disease progression after exposure to adequate chemotherapy dose intensity that was adjudicated independently.

It is hypothesised that panitumumab induced clinical benefit by halting disease progression. A similar effect has been observed for other targeted compounds used in monotherapy (Ratain *et al*, 2006; Escudier *et al*, 2007). Therefore, we then evaluated the association of PFS and clinical outcomes in patients with mCRC refractory to standard chemotherapy regimens. There was a significant and clinically meaningful association between being progression free and better HRQoL, CRC symptomatology, and OS in panitumumab patients. An association between improved PFS and favourable OS prognosis was also observed in the BSC arm, potentially reflecting a subpopulation with more indolent disease. However, compared to BSC alone, treatment with panitumumab resulted in approximately 20% more patients who were progression free at weeks 8, 12, and 16 (Van Cutsem *et al*, 2007a, 2007b). Although more patients were rendered progression free with panitumumab compared to BSC, and being progression free was associated with a favourable OS prognosis, these progression-free rate differences did not translate into differences in OS when all patients were compared (Van Cutsem *et al*, 2007a, 2007b), possibly due to the effect of early crossover of patients in the BSC to panitumumab treatment. Indeed, 76% of patients in the BSC arm crossed over to receive panitumumab at a median time of 7.0 weeks. In these patients, panitumumab treatment was associated with similar clinical activity as that in the phase 3 study (Van Cutsem *et al*, 2007a, 2007b).

The reasons underlying the association between progression status and HRQoL in the panitumumab arm may relate to the overall effects of panitumumab on tumour growth arrest. Although objective responses were observed in 10% of patients, a larger number of patients within the stable disease population also had reductions in tumour burden. Indeed, in panitumumab patients with stable disease, there was a median decrease in maximum target lesion size of approximately 12% compared to baseline, whereas BSC patients with stable disease had a median increase of approximately 7% (Amgen, data on file). Overall, the majority of patients achieving a best response of at least stable disease in the panitumumab group had a reduction in tumour volume, which was associated with symptom improvement.

Missing data for QoL end points was largely due to disease progression (and therefore QoL data collection halted) in this advanced-stage population, especially among the BSC patients, 70% of whom had disease progression by week 8 (and subsequently crossed over to panitumumab therapy in the extension trial). By using either of the two imputation methods, LVCF and slope, the true effect on the PROs by treatment may have been underestimated. With the LVCF method, imputation of data halts progression at dropout or crossover and carries forward the pre-dropout assessment throughout the study, with zero carried forward at death. This method may bias the results in favour of a treatment that results in more dropouts associated with morbidity. In this case it can result in bias by imputing potentially higher scores in the BSC patient than the panitumumab patients. In some settings, the slope method may be more conservative and less biased towards either treatment group, and imputation of a score of zero at death with the LVCF method may be biased against groups with high death rates. These limitations do not seem to have affected the overall findings as results were consistent across imputation methods. However, these imputation methods ignore the effect of panitumumab received after crossover on QoL measures, as these end points were not measured in the crossover extension trial.

These results further suggest that halting disease progression is associated with clinical benefit of sustained or improved QoL and prolonged survival in patients with chemorefractory mCRC. This hypothesis should be tested prospectively in randomised-controlled trials. Further studies are ongoing to evaluate the benefits of panitumumab in subsets of patients with different molecular characteristics of resistance or sensitivity to this targeted therapeutic agent (Moroni *et al*, 2005; Lievre *et al*, 2006; Benvenuti *et al*, 2007; Sartore-Bianchi *et al*, 2007) as well as in earlier lines of therapy.

## Figures and Tables

**Table 1:** PRO assessment and schedule

**PRO instrument**	**Assessment schedule**
FACT-FCSI	Baseline, every 2 weeks during treatment, and at the 30-day safety follow-up visit
EUROQOL (EQ-5D) Health Index	Baseline, monthly during treatment, and at the 30-day safety follow-up visit
EUROQOL (EQ-5D) Visual Analog Scale (VAS)	
EORTC QLQ-C30 Global Health Status/QoL Scale	

**Table 2:** Patient analysis sets and PRO data availability

	**Panitumumab plus BSC**	**BSC alone**
Total randomised (*n*)	231	232
PRO all enrolled analysis set (*n*)	207	184

PRO all enrolled analysis set and alive at week 8, EQ-5D (*n*)	179	164
*Patients completing EQ-5D* (*n*)		
Week 4	189	129
Week 8	111	47
Week 12	91	14
Week 16	62	7

PRO all enrolled analysis set and alive at week 8, FCSI (*n*)	181	166
*Patients completing FCSI subscale* (*n*)		
Week 4	190	130
Week 8	112	48
Week 12	90	14
Week 16	62	7
